# Psychosocial and lifestyle impacts of spontaneous coronary artery dissection: A quantitative study

**DOI:** 10.1371/journal.pone.0296224

**Published:** 2024-01-05

**Authors:** Barbara M. Murphy, Michelle C. Rogerson, Michael R. Le Grande, Stephanie Hesselson, Siiri E. Iismaa, Robert M. Graham, Alun C. Jackson

**Affiliations:** 1 Australian Centre for Heart Health, Melbourne, Victoria, Australia; 2 School of Psychological Sciences, University of Melbourne, Melbourne, Victoria, Australia; 3 Victor Chang Cardiac Research Institute, Sydney, New South Wales, Australia; 4 St Vincent’s Hospital, Sydney, New South Wales, Australia; 5 Centre on Behavioral Health, University of Hong Kong, Pokfulam, Hong Kong; University of Roehampton - Whitelands College, UNITED KINGDOM

## Abstract

**Introduction:**

Recent studies suggest that acute myocardial infarction due to spontaneous coronary artery dissection (SCAD) carries significant psychosocial burden. This survey-based quantitative study builds on our earlier qualitative investigation of the psychosocial impacts of SCAD in Australian SCAD survivors. The study aimed to document the prevalence and predictors of a broad range of psychosocial and lifestyle impacts of SCAD.

**Method:**

Australian SCAD survivors currently enrolled in the Victor Chang Cardiac Research Institute genetics study were invited to participate in an online survey to assess the psychosocial impacts of SCAD. Participants completed a questionnaire, developed using findings from our earlier qualitative research, which assessed 48 psychosocial and five lifestyle impacts of SCAD. Participants also provided demographic and medical data and completed validated measures of anxiety and depression.

**Results:**

Of 433 SCAD survivors invited to participate, 310 (72%) completed the questionnaire. The most common psychosocial impacts were ‘shock about having a heart attack’ (experienced by 87% respondents), ‘worry about having another SCAD’ (81%), ‘concern about triggering another SCAD’ (77%), ‘uncertainty about exercise and physical activity’ (73%) and ‘confusion about safe levels of activity and exertion’ (73.0%) and ‘being overly aware of bodily sensations’ (73%). In terms of lifestyle impacts, the SCAD had impacted on work capacity for almost two thirds of participants, while one in ten had sought financial assistance. The key predictors of psychosocial impacts were being under 50, current financial strain, and trade-level education. The key predictors of lifestyle impacts were being over 50, SCAD recurrence, trade-level education, and current financial strain. All psychosocial impacts and some lifestyle impacts were associated with increased risk of anxiety and/or depression.

**Conclusion and implications:**

This quantitative study extends our previous qualitative investigation by documenting the prevalence of each of 48 psychosocial and five lifestyle impacts identified in our earlier focus group research, and by providing risk factors for greater SCAD impacts. The findings suggest the need for supports to address initial experiences of shock, as well as fears and uncertainties regarding the future, including SCAD recurrence and exercise resumption. Support could be targeted to those with identified risk factors. Strategies to enable SCAD survivors to remain in or return to the paid workforce are also indicated.

## Introduction

Research into the psychosocial impacts of spontaneous coronary artery dissection (SCAD) has gained increased momentum in recent years. The stressfulness of SCAD-related acute myocardial infarction (AMI) is due to its unexpected occurrence and sudden onset, unclear cause and uncertain management, together with its relatively high recurrence rate [1, 2]. Predominantly affecting younger women without atherosclerosis and with few traditional cardiac risk factors [3–5], SCAD comes as a shock and is not suited to traditional lifestyle management approaches [6, 7]. Emotional stress is often reported as a precipitator to SCAD events [4, 6, 8–11].

Several studies undertaken in the US, Canada, Australia, and Europe have documented relatively high rates of anxiety and depression in the aftermath of SCAD [11–17]. Emerging evidence suggests that SCAD-AMI may be more stressful than typical atherosclerotic AMI, with higher rates of post-event anxiety and depression [13, 17]. However, while previous quantitative studies focus on rates of anxiety, depression and stress, they do not describe the exact nature of specific concerns experienced by SCAD survivors.

To understand the concerns and emotional impacts experienced by SCAD survivors, we undertook and reported on a qualitative study involving 30 SCAD survivors [18]. We found that SCAD survivors experienced a broad range of psychosocial impacts, including confusion and uncertainty about what SCAD was and how to manage it; feelings of shock, sadness, vulnerability, frustration, unfairness, guilt, embarrassment, isolation and loneliness; loss of self-confidence and reduced sense of self; fear about the future including concerns about recurrence and death; concern, confusion, frustration and helplessness in efforts to manage SCAD; negative impacts on roles and relationships, including work restrictions and family stress; and feeling dismissed by health professionals and abandoned by the health system [18]. The findings of the qualitative study demonstrated that the emotions experienced by SCAD survivors are complex, inter-related, and overwhelming [18]. The findings also demonstrated that SCAD impacted on survivors’ work life, financial status, and engagement in sport and exercise [18]. Following this qualitative work, a quantitative study was required to assess the extent of these psychosocial and lifestyle impacts, to identify those most at risk of experiencing them, and to consider their impacts on SCAD survivors’ emotional wellbeing.

### Aims of the present study

The present study extends our earlier qualitative work by seeking to assess the extent and predictors of the psychosocial and lifestyle impacts of SCAD through an online survey of SCAD survivors. The aims of the study were threefold: First, to quantify each of the psychosocial and lifestyle impacts of SCAD identified in our qualitative study, thereby assessing their prevalence and relative importance. Second, to identify the characteristics of SCAD survivors who are most likely to experience these impacts. And third, to investigate the association between the SCAD impacts and survivors’ current anxiety and depression.

## Method

### Governance

The study was approved by the Human Research Ethics Committee (HREC) of St Vincent’s Hospital Sydney (HREC/16/SVH/338).

### Participants and recruitment

Participants were recruited by the Victor Chang Cardiac Research Institute from a database of SCAD survivors participating in a larger genetic study (the VC Genetics Study) [19]. The VC Genetics Study currently involves 433 participants aged over 18 who have had at least one SCAD event, with no exclusion based on time since SCAD. All participants have adequate English language proficiency to read and understand the consent form and study information. No additional exclusions were imposed for the current study. This was the same database from which participants were recruited for our earlier qualitative study [18].

To recruit participants for the online survey, two separate sub-databases were created by the VC Genetics Study Coordinator (SH), one comprising participants whose most recent SCAD had been confirmed on angiogram by the VC cardiologist, and one comprising participants whose most recent SCAD had not been confirmed on angiogram by the VC cardiologist, either because of delays in accessing the angiogram (due to recency of the event) or inability to acquire the angiogram. During recruitment, the two databases were kept separate to facilitate separate data cleansing of responses. The ‘confirmed’ database comprised 347 SCAD survivors, while the ‘not yet confirmed’ database comprised 86 SCAD survivors, a combined total of 433.

### Procedure

The recruitment period began on 10 February 2023 and ended on 6 May 2023. Each of the 433 potential participants was emailed a unique link to access the Participant Information and Consent Form (PICF). After providing written consent by ticking the ‘consent’ box to indicate that they had read and understood the PICF, participants accessed the online questionnaire available via the Research Electronic Data Capture (REDCap) platform. The questionnaire took approximately 25 minutes to complete. No identifying information was collected as no participant follow-up was involved. All respondent data were collected by self-report.

### Measures

#### Confirmation of SCAD

Respondents in both the ‘confirmed’ and ‘not yet confirmed’ samples were asked two self-report items to assist in confirming that they had had SCAD: 1) have you been told by a health professional that you have had SCAD, and 2) has your SCAD been confirmed by angiogram.

#### Sociodemographic and medical questions

Sociodemographic information included sex, age in years, partner status, employment status, education, private health insurance, financial strain (5-point scale from none to severe), living arrangements, presence of a close confidante, and loss of a close relative or friend in the past 12 months (defined as ‘recent bereavement’). Age in years was categorised as ≤50 vs. 51–60 vs. >60. Medical information included number of SCADs, time since most recent SCAD, SCAD-specific risk factors (fibromuscular dysplasia [FMD], migraine headache, connective tissue disorder, inflammatory disorders [1, 4, 8, 9, 20]), traditional cardiac risk factors (high blood pressure, high cholesterol, diabetes, gestational diabetes, obesity, sleep apnoea, cigarette smoking), comorbidities (musculoskeletal conditions, stroke, cancer), and SCAD treatments (medications and procedures). Participants self-reported on whether they had a history of a mental health condition prior to their SCAD, namely history of anxiety, depression, and/or post-traumatic stress disorder (PTSD). Responses were combined to provide a single item relating to mental health history. Participants reported on whether they had attended a cardiac rehabilitation (CR) program or other support programs.

#### SCAD impact measures

*SCAD psychosocial impacts*. Based on the findings of our earlier qualitative study, a set of 48 items was generated to assess the psychosocial impacts of SCAD. Respondents were asked to indicate how much they had experienced each impact during the first 6 months after SCAD. Each item was rated on a 4-point scale where 0 = not at all, 1 = a small amount, 2 = a moderate amount, and 3 = a large amount. *SCAD lifestyle impacts*. Also based on qualitative findings, five items asked about lifestyle impacts due to SCAD, including stopping work, reducing work hours, changing jobs, seeking financial support, and ceasing doing favorite sport or exercise. Respondents were asked to indicate whether SCAD had resulted in each impact. Each item was rated as 0 = no or 1 = yes.

#### Measures of current anxiety and depression

Validated instruments were included to assess respondents’ current anxiety and depression in the two weeks prior to study participation. *Anxiety*. Anxiety was assessed using the Generalised Anxiety Disorder 7-item scale (GAD7) [21]. GAD7 scores ≥10 were used to indicate the presence of current anxiety. The GAD7 has good reliability and validity for detecting generalized anxiety [21], and has been well validated in the general population [22] and with cardiac patients [23]. *Depression*. Depression was assessed using the 9-item Patient Health Questionnaire (PHQ9) [24]. PHQ9 scores ≥10 were used to indicate the presence of current depression. The PHQ9 has been validated with cardiac patients [25] and has been endorsed by the National Heart Foundation of Australia as the recommended tool for depression screening in cardiac populations [26].

### Data cleansing

Unidentified data from RedCap were saved to the statistical program (SPSSx) for data analysis. Data cleansing was undertaken to remove individuals from the ‘not yet confirmed’ sample who did not clearly have confirmed SCAD, that is, those who did not indicate on self-report that they had been told they had SCAD (*n* = 0 cases) or that their SCAD had been confirmed by an angiogram (*n* = 7 cases). Data cleansing was also undertaken to remove repeat entries from the same individual; repeat entries were identified as cases with exact entries for postcode, sex, age, living arrangements, marital status, work status, time since SCAD, and number of SCADs. In each case, the most complete entry was retained or, in cases where more than one entry was complete, the first entry was retained. In total, 31 repeat cases were deleted. The final sample comprised 310 eligible participants, 250 (81%) from the ‘confirmed’ sample and 60 (19%) from the ‘not yet confirmed’ sample. All retained cases indicated that they had been told by a health professional that they had SCAD and that their SCAD had been confirmed on an angiogram. There were no differences between the remaining ‘confirmed’ and ‘not yet confirmed’ sub-samples on any of the sociodemographic or clinical variables, or on the validated psychosocial instruments, thus the two sub-samples were combined for the analyses.

### Data analysis

#### Prevalence of SCAD impacts

Frequencies were calculated to identify participant endorsement of each SCAD psychosocial and lifestyle impact item. The 48 psychosocial impact items were grouped *a priori* into eight domains to assess: 1) emotional impacts (9 items); 2) concerns about the future (5 items); 3) difficulties managing SCAD (7 items); 4) uncertainty and confusion (5 items); 5) impacts on relationships (6 items); 6) impacts on self-perception (4 items); 7) impacts on roles (7 items); and 8) impacts of the health system (5 items). An average scale score was calculated for each domain by dividing ratings across domain items by the number of items per domain.

#### Predictors of SCAD impacts

Bivariate analyses (t-tests and analysis of variance for psychosocial domains and chi-square tests for lifestyle ratings) were used to identify associations with each of the sociodemographic, medical and psychosocial participant characteristics shown in Table 1. All variables showing statistical (*p* ≤ 0.2) or conceptual importance were retained for the multivariate analyses, except in cases where there was collinearity with another variable. At this first step, the conservative *p* value of ≤ 0.2 was used to minimize the possibility of Type 2 error. Multivariate linear regression was used to identify significant predictors of psychosocial domain scores, whereas multivariate logistic regression was used to identify significant predictors of lifestyle ratings. McKelvey and Zavoina’s pseudo R-squared to estimate explained variance of the logistic regression models [27] was used since it performs closest to the ordinary least squares R-squared in simulation studies [28]. At this second step, the Benjamini-Hochberg Adjusted *p* value was calculated and applied to account for multiple comparisons, thereby minimizing the possibility of Type 1 error.

**Table 1:** Characteristics of participants.

		N	n	%
***Sociodemographic characteristics***				
Sex	Female	310	295	95.2
	Male		14	4.5
	Prefer not to say		1	0.3
Current age	50 or younger	310	84	27.1
	51–60		134	43.2
	Over 60 years		92	29.7
Country of birth	Australia	310	243	78.4
	Outside Australia		67	21.6
Partner status	Married/partnered	309	246	79.6
	Divorced/separated		37	12.0
	Widowed		9	2.9
	Never married		17	5.5
Education	Secondary	310	43	13.9
	Trade or certificate		69	22.3
	University degree/post-graduate		198	63.9
Employment status	Employed	310	256	82.6
	Unemployed		6	1.9
	Not in paid workforce		48	15.5
Insurance status	Have health insurance	310	236	76.1
	No health insurance		74	23.9
***Psychosocial characteristics***				
Lives alone		310	44	14.2
No close confidante		310	35	11.3
Recent bereavement ^a^		310	67	21.6
Financial strain		310	94	30.3
Current anxiety (GAD7 ≥10)^b^		290	60	20.7
Current depression (PHQ9 ≥10) ^b^		287	60	20.9
Mental health history prior to SCAD		310	97	31.3
***Medical characteristics***				
Time since last SCAD^c^	Within previous year	310	42	13.5
	1–3 years ago		84	27.1
	3–5 years ago		69	22.3
	5+ years ago		115	37.1
Total number SCADs	One	310	241	77.7
	Two		46	14.8
	Three		16	5.2
	Four		4	1.3
	Five		3	1.0
SCAD treatments	Medication	310	256	82.6
	Percutaneous coronary intervention	310	42	13.5
	Coronary artery bypass surgery	310	6	1.9
	Implantable cardioverter defibrillator	310	3	1.0
SCAD-specific risk factors	Migraine	310	102	32.9
	Fibromuscular dysplasia	310	53	17.1
	Inflammatory disorder	310	21	6.8
	Connective tissue disorder	310	5	1.6
Traditional cardiac risk factors	High blood pressure	310	92	29.7
	High cholesterol	310	39	12.6
	Obesity	310	33	10.6
	Sleep apnoea	310	28	9.0
	Diabetes	310	8	2.6
	Gestational diabetes	310	5	1.6
	Cigarette smoking	310	6	1.9
Cormorbidities	Musculoskeletal condition	310	54	17.4
	Stroke or Transient Ischemic Attack	310	8	2.6
	Cancer	310	4	1.3
***Supports after SCAD***				
	Attended cardiac rehabilitation ^c^	310	195	62.9
	Joined Facebook support group	310	194	62.6
	Attended online support group	310	19	6.1
	Attended face-to-face support group	310	9	2.9

N = 310.

^a^ in past 12 months

^b^ in past two weeks

^c^ since most recent SCAD. Recent bereavement = having lost a relative or friend in the past 12 months. Financial strain = reports of moderate, considerable or extreme financial strain. PTSD = post-traumatic stress disorder; GAD7 = Generalised Anxiety Disorder 7-item; PHQ9 = Patient Health Questionnaire 9-item; Mental health history = reports of history of anxiety, depression or post-traumatic stress disorder; SCAD = spontaneous coronary artery dissection.

#### Association between SCAD impacts and current anxiety and depression

T-tests and chi-square tests were used to compare psychosocial impact domain scores and lifestyle impact ratings for anxious vs. not anxious, and depressed vs. not depressed respondents. Again the Benjamini-Hochberg Adjusted *p* value was calculated and applied to account for multiple comparisons, thereby minimizing the possibility of Type 1 error.

### Anticipated sample size and study power

From the study population of 433 SCAD survivors, it was anticipated that approximately 300 would participate. This sample size is larger than previous studies of anxiety and depression in SCAD survivors [12–14] and was deemed adequate for the required analyses.

## Results

### Participant characteristics

#### Sociodemographic and medical characteristics

A total of 310 participants was included, representing a 72% response rate from the population of 433 SCAD survivors invited to participate. Participants’ ages ranged from 30–77 (mean = 55.61, SD = 9.07). Most were born in Australia, with others born in the UK (*n* = 21, 7%), New Zealand (*n* = 17, 6%), South Africa (*n* = 4, 1%), Malaysia (*n* = 3, 1%) and Ireland (*n* = 3, 1%). Two identified as being Aboriginal or Torres Strait Islander (Indigenous Australian). In terms of psychological characteristics, 21% were classified as anxious (GAD7≥10) and 21% were classified as depressed (PHQ9≥10). Other sociodemographic and clinical characteristics are shown in Table 1.

### Impacts of SCAD

#### Psychosocial impacts

**Fig 1:**
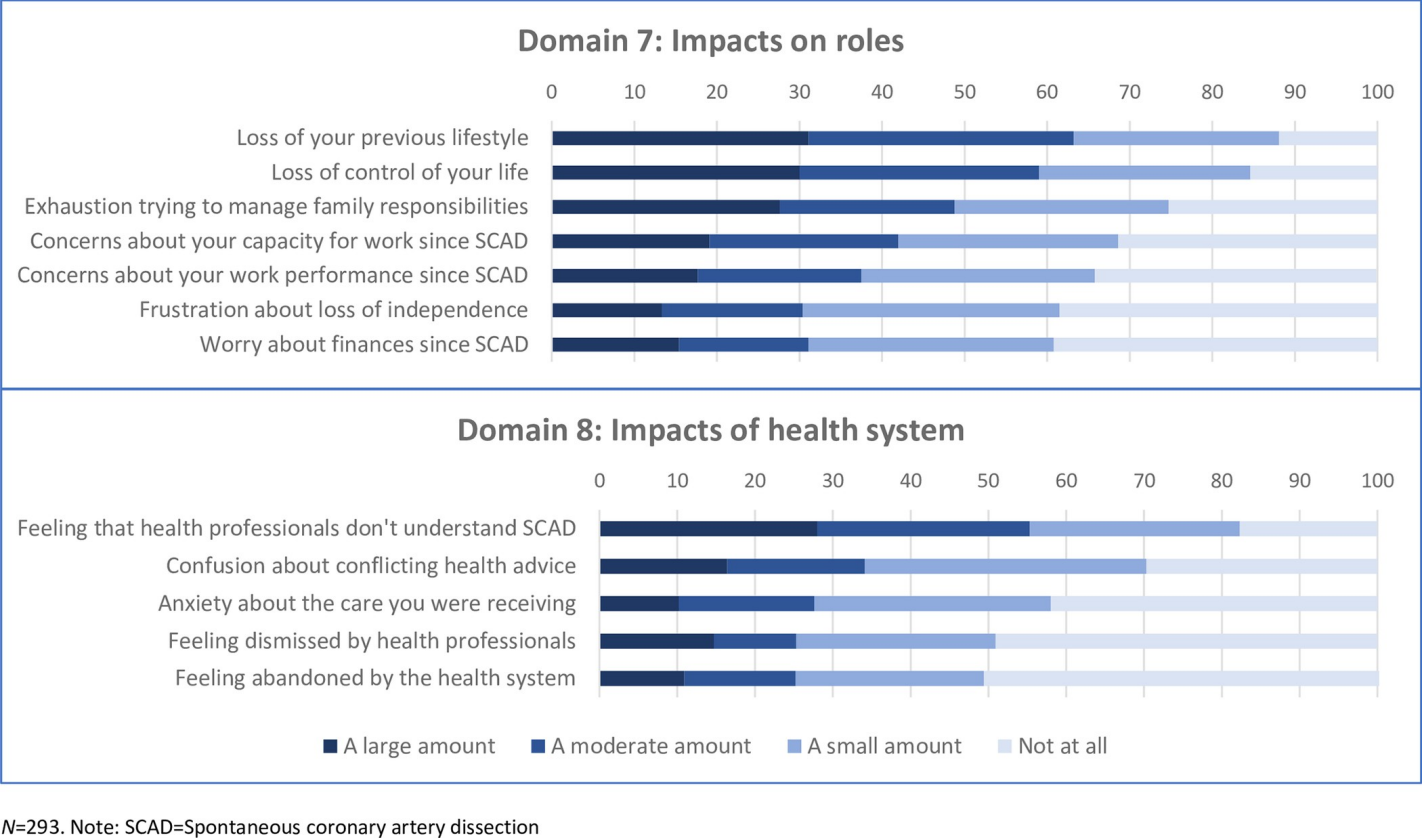


Ratings for all SCAD psychosocial impact items across the eight domains are shown in Fig 1. In interpreting the figures, responses of ‘a large amount’ or ‘a moderate amount’ are combined to differentiate the most highly endorsed items.

The most highly endorsed item was ‘shock about having a heart attack’, from the *Emotional Impacts* domain (Fig 1), which was experienced by almost all respondents (86.5% a large or moderate amount). Only 2% of respondents did not experience shock. Other commonly endorsed *Emotional Impacts* were ‘sadness about having had SCAD’ (63.5% a large or moderate amount), and ‘feeling vulnerable’ (53.9%).

In the *Future Concerns* domain (Fig 1), ‘worry about having another SCAD’ was experienced by almost all respondents (80.5% a large or moderate amount), with only 2.4% not experiencing this worry. Over two thirds experienced ‘fear about the future’ (69.7% a large or moderate amount), and the remaining three items were largely or moderately endorsed by more than half the respondents: ‘uncertainty about life ahead’ (56.6%), ‘fear of dying’ (54.6%) and ‘concerns about whether life will return to normal’ (50.5%).

In the *Difficulties with Management* domain (Fig 1), ‘concern about triggering another SCAD’ was experienced by over three quarters of respondents (76.8% a large or moderate amount). Only 4.4% did not experience this concern. Other commonly experienced concerns were ‘being overly aware of bodily sensations’ (73.0% a large or moderate amount), ‘concerns about side effects of medications’ (62.4%); ‘frustration about fatigue and tiredness’ (59.3%) and ‘lack of control in effectively managing the condition’ (53.9%)

In the *Uncertainty and Confusion* domain (Fig 1), around three quarters of respondents largely or moderately endorsed ‘uncertainty about exercise and physical activity’ (73.1%) and ‘confusion about safe levels of activity and exertion’ (73.0%). A further two thirds indicated experiencing ‘uncertainty about managing your health condition’ (64.9% a large or moderate amount).

In the *Impacts on Relationships* domain (Fig 1), around two thirds of respondents experienced ‘worry about impacts of your SCAD on family members’ (69.9% a large or moderate amount). In the *Self-perception domain* (Fig 1), around two thirds experienced ‘loss of self-confidence’ (63.8% a large or moderate amount) and ‘feeling let down by your body’ (62.1%), and around half experienced concern about weight gain’ (54.3%). In the *Impacts on Roles* domain (Fig 1), the most common experiences were ‘loss of your previous lifestyle’ (63.2%) and ‘loss of control of your life’ (59.0%). In the *Impacts of Health System* domain (Fig 1), the most commonly endorsed item was ‘feeling that health professionals don’t understand SCAD’ (55.3%).

#### Lifestyle impacts

**Fig 2:**
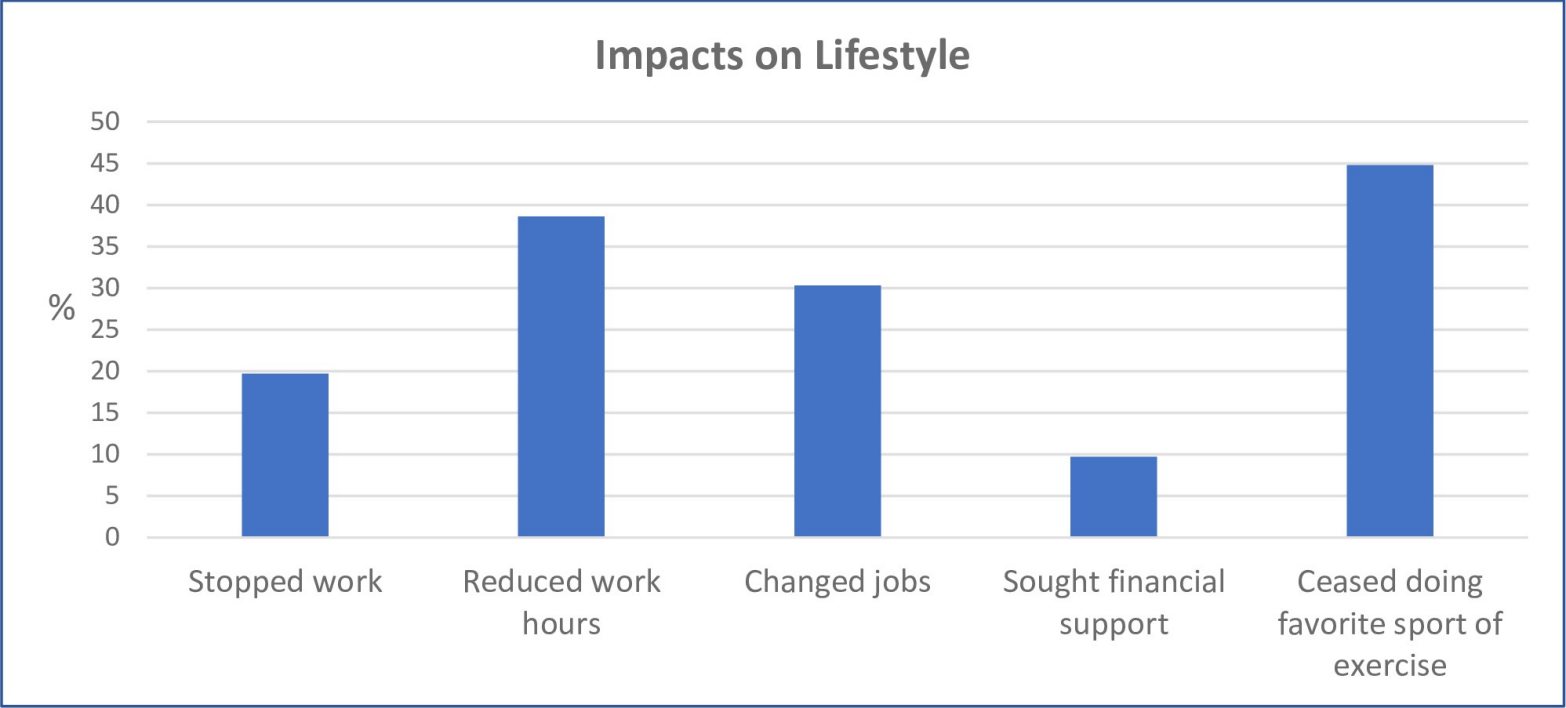
Proportions experiencing each lifestyle impact. N = 293.

Responses regarding impacts of SCAD on lifestyle are shown in Fig 2.

As shown in Fig 2, almost half the respondents had ceased doing a favorite sport or exercise. In terms of impacts of SCAD on work, over a third had reduced their work hours, almost a third had changed jobs, and approximately one-fifth had stopped work altogether. Combined, 176 participants (60.1%) reported at least one of these work impacts. Around one in ten had sought financial assistance.

### Predictors of SCAD Impacts

#### Psychosocial impacts

The variables retained on statistical grounds for inclusion in the linear regression to predict psychosocial impacts were age, education, financial strain, recent bereavement, presence of close confidante, health insurance status, and smoking status. The variables retained on conceptual grounds were mental health history, number of SCADs (dichotomized as one vs. two or more), time since SCAD, and CR attendance. The variables that did not qualify for inclusion in the multivariate analyses were sex, country of birth, partner status, employment status, living alone, and each of the SCAD treatments, comorbidities, and CVD risk factors. Results of the multivariable linear regression are shown in Table 2.

**Table 2:** Predictors of SCAD psychosocial impacts domain scores.

	**Emotional impacts**				**Concerns about the future**				**Difficulties managing SCAD**			
	Beta	95%CI	β	*p*	Beta	95%CI	β	*p*	Beta	95%CI	β	*p*
**Age group**												
30–50	4.07	[2.21,5.93]	0.29	**<0.001**	2.36	[1.11,3.61]	0.26	**<0.001**	3.02	[1.44,4.60]	0.26	**<0.001**
51–60	1.96	[0.23,3.68]	0.15	0.027	1.02	[-0.18,2.21]	0.13	0.097	1.40	[-0.15,2.95]	0.13	0.076
61+	Ref		0.00	.	Ref		0.00	.	Ref		0.00	.
**Financial strain**												
No	Ref		0.00	.	Ref		0.00	.	Ref		0.00	.
Yes	2.45	[0.89,4.01]	0.18	**0.002**	1.50	[0.44,2.56]	0.17	**0.006**	2.09	[0.78,3.40]	0.18	**0.002**
**Education**												
Secondary	1.57	[-0.56,3.70]	0.08	0.148	0.42	[-0.89,1.74]	0.04	0.527	1.02	[-0.76,2.80]	0.07	0.260
Trade or TAFE	2.39	[0.73,4.05]	0.16	**0.005**	1.35	[0.26,2.44]	0.14	**0.015**	1.67	[0.26,3.08]	0.13	**0.020**
University	Ref		0.00	.	Ref		0.00	.	Ref		0.00	.
**Health insurance**												
Uninsured	Ref		0.00	.	Ref		0.00	.	Ref		0.00	.
Insured	0.85	[-0.85,2.55]	0.06	0.325	1.18	[0.13,2.23]	0.12	0.027	0.62	[-0.67,1.92]	0.05	0.344
**Smoking status**												
Never smoked	Ref		0.00	.	Ref		0.00	.	Ref		0.00	.
Current or former	1.06	[-1.04,3.17]	0.06	0.320	0.76	[-0.49,2.01]	0.07	0.231	0.74	[-0.85,2.33]	0.05	0.359
**Recent bereavement**												
No	Ref		0.00	.	Ref		0.00	.	Ref		0.00	.
Yes	1.62	[0.06,3.19]	0.11	0.042	0.71	[-0.34,1.76]	0.07	0.184	1.20	[-0.16,2.56]	0.10	0.083
**Have confidante**												
No	1.28	[-0.97,3.53]	0.07	0.265	0.28	[-1.28,1.83]	0.02	0.727	0.74	[-1.27,2.74]	0.05	0.469
Yes	Ref		0.00	.	Ref		0.00	.	Ref		0.00	.
**Mental health history**												
No	Ref		0.00	.	Ref		0.00	.	Ref		0.00	.
Yes	-1.15	[-2.66,0.37]	-0.09	0.136	-0.43	[-1.45,0.60]	-0.05	0.415	-0.51	[-1.77,0.74]	-0.05	0.420
**Number of SCADs**												
One	Ref		0.00	.	Ref		0.00	.	Ref		0.00	.
Two or more	1.11	[-0.60,2.81]	0.07	0.201	0.56	[-0.51,1.63]	0.06	0.302	0.96	[-0.41,2.33]	0.08	0.170
**Time since SCAD**												
In previous year	Ref		0.00	.	Ref		0.00	.	Ref		0.00	.
13–36 months ago	1.26	[-1.17,3.68]	0.09	0.309	0.05	[-1.42,1.52]	0.01	0.949	0.05	[-1.84,1.94]	0.01	0.962
37–60 months ago	0.94	[-1.59,3.46]	0.06	0.466	0.07	[-1.56,1.70]	0.01	0.932	-0.15	[-2.18,1.87]	-0.01	0.881
Over 5 years ago	2.03	[-0.44,4.50]	0.16	0.107	0.81	[-0.70,2.32]	0.10	0.293	0.45	[-1.47,2.37]	0.04	0.647
**Attend CR**												
No	Ref		0.00	.	Ref		0.00	.	Ref		0.00	.
Yes	1.05	[-0.31,2.42]	0.08	0.130	-0.14	[-1.07,0.79]	-0.02	0.765	0.67	[-0.52,1.86]	0.06	0.267
Observations	293				293				293			
R2	0.160				0.124				0.132			
	**Uncertainty and confusion**				**Impacts on relationships**				**Impacts on self-perception**			
	Beta	95%CI	β	p	Beta	95%CI	β	p	Beta	95%CI	β	p
**Age group**												
30–50	1.85	[0.68,3.02]	0.23	**0.002**	3.39	[2.07,4.71]	0.36	**<0.001**	1.78	[0.76,2.81]	0.25	**0.001**
51–60	1.01	[-0.10,2.11]	0.14	0.075	1.24	[0.14,2.34]	0.14	0.028	1.01	[0.14,1.88]	0.16	0.023
61+	Ref		0.00	.	Ref		0.00	.	Ref		0.00	.
**Financial strain**												
No	Ref		0.00	.	Ref		0.00	.	Ref		0.00	.
Yes	0.98	[0.05,1.91]	0.12	**0.039**	1.89	[0.81,2.97]	0.20	**0.001**	1.09	[0.30,1.87]	0.16	**0.007**
**Education**												
Secondary	0.43	[-0.78,1.64]	0.04	0.488	1.44	[0.02,2.85]	0.11	0.046	0.48	[-0.56,1.52]	0.05	0.362
Trade or TAFE	1.10	[0.08,2.12]	0.13	**0.035**	1.82	[0.67,2.97]	0.18	**0.002**	1.14	[0.24,2.03]	0.15	**0.013**
University	Ref		0.00	.	Ref		0.00	.	Ref		0.00	.
**Health insurance**												
Uninsured	Ref		0.00	.	Ref		0.00	.	Ref		0.00	.
Insured	0.45	[-0.51,1.41]	0.05	0.359	0.05	[-1.05,1.15]	0.01	0.925	0.28	[-0.56,1.11]	0.04	0.517
**Smoking status**												
Never smoked	Ref		0.00	.	Ref		0.00	.	Ref		0.00	.
Current or former	0.69	[-0.52,1.89]	0.07	0.261	1.33	[-0.12,2.78]	0.11	0.072	0.61	[-0.44,1.66]	0.07	0.255
**Recent bereavement**												
No	Ref		0.00	.	Ref		0.00	.	Ref		0.00	.
Yes	0.82	[-0.14,1.78]	0.09	0.094	0.47	[-0.57,1.51]	0.05	0.376	0.83	[0.05,1.61]	0.11	0.037
**Have confidante**												
No	0.47	[-0.95,1.90]	0.04	0.514	1.17	[-0.26,2.60]	0.09	0.108	0.31	[-0.78,1.39]	0.03	0.579
Yes	Ref		0.00	.	Ref		0.00	.	Ref		0.00	.
**Mental health history**												
No	Ref		0.00	.	Ref		0.00	.	Ref		0.00	.
Yes	-0.84	[-1.73,0.06]	-0.11	0.068	-0.14	[-1.12,0.85]	-0.02	0.785	0.08	[-0.69,0.85]	0.01	0.837
**Number of SCADs**												
One	Ref		0.00	.	Ref		0.00	.	Ref		0.00	.
Two or more	-0.14	[-1.13,0.85]	-0.02	0.778	1.16	[0.12,2.20]	0.11	0.029	0.68	[-0.19,1.54]	0.09	0.126
**Time since SCAD**												
In previous year	Ref		0.00	.	Ref		0.00	.	Ref		0.00	.
13–36 months ago	0.70	[-0.66,2.06]	0.09	0.313	0.50	[-0.97,1.97]	0.05	0.506	0.40	[-0.74,1.55]	0.06	0.488
37–60 months ago	0.37	[-1.08,1.82]	0.04	0.613	0.12	[-1.35,1.60]	0.01	0.868	-0.09	[-1.29,1.10]	-0.01	0.881
Over 5 years ago	0.66	[-0.71,2.03]	0.09	0.343	0.47	[-0.93,1.88]	0.05	0.509	0.30	[-0.85,1.45]	0.05	0.608
**Attend CR**												
No	Ref		0.00	.	Ref		0.00	.	Ref		0.00	.
Yes	0.37	[-0.50,1.24]	0.05	0.406	0.93	[0.01,1.84]	0.11	0.046	0.41	[-0.28,1.10]	0.06	0.243
Observations	293				293				293			
R2	0.098				0.232				0.138			

	**Impacts on roles**				**Impacts of health system**			
	Beta	95%CI	β	p	Beta	95%CI	β	p
**Age group**								
30–50	3.85	[2.14,5.56]	0.30	**<0.001**	1.00	[-0.43,2.43]	0.10	0.171
51–60	1.67	[0.09,3.25]	0.14	0.038	0.38	[-0.76,1.52]	0.04	0.508
61+	Ref		0.00	.	Ref		0.00	.
**Financial strain**								
No	Ref		0.00	.	Ref		0.00	.
Yes	3.08	[1.68,4.48]	0.24	**<0.001**	1.64	[0.50,2.77]	0.17	**0.005**
**Education**								
Secondary	-0.34	[-2.14,1.47]	-0.02	0.713	0.17	[-1.49,1.83]	0.01	0.839
Trade or TAFE	2.00	[0.45,3.55]	0.14	**0.012**	1.21	[-0.05,2.47]	0.12	0.060
University	Ref		0.00	.	Ref		0.00	.
**Health insurance**								
Uninsured	Ref		0.00	.	Ref		0.00	.
Insured	0.84	[-0.51,2.19]	0.06	0.223	-0.29	[-1.49,0.91]	-0.03	0.636
**Smoking status**								
Never smoked	Ref		0.00	.	Ref		0.00	.
Current or former	0.54	[-1.19,2.27]	0.03	0.540	1.42	[-0.11,2.96]	0.12	0.068
**Recent bereavement**								
No	Ref		0.00	.	Ref		0.00	.
Yes	1.15	[-0.31,2.61]	0.08	0.122	-0.10	[-1.23,1.02]	-0.01	0.859
**Have confidante**								
No	1.20	[-0.92,3.32]	0.07	0.266	0.90	[-0.64,2.44]	0.07	0.251
Yes	Ref		0.00	.	Ref		0.00	.
**Mental health history**								
No	Ref		0.00	.	Ref		0.00	.
Yes	0.05	[-1.27,1.37]	0.00	0.942	0.19	[-0.86,1.24]	0.02	0.722
**Number of SCADs**								
One	Ref		0.00	.	Ref		0.00	.
Two or more	1.31	[-0.13,2.75]	0.09	0.074	0.69	[-0.55,1.93]	0.07	0.276
**Time since SCAD**								
In previous year	Ref		0.00	.	Ref		0.00	.
13–36 months ago	-0.63	[-2.73,1.47]	-0.05	0.553	0.68	[-1.07,2.42]	0.07	0.446
37–60 months ago	-1.18	[-3.28,0.92]	-0.09	0.270	0.09	[-1.64,1.82]	0.01	0.921
Over 5 years ago	-0.42	[-2.51,1.68]	-0.04	0.696	0.56	[-1.11,2.23]	0.06	0.509
**Attend CR**								
No	Ref		0.00	.	Ref		0.00	.
Yes	0.87	[-0.40,2.15]	0.07	0.179	-0.93	[-1.92,0.06]	-0.11	0.067
Observations	293				293			
R2	0.206				0.101			

Note. Full multivariable linear regression models for each rating included: age (under 50 vs. 51–60 vs. 61+), financial stress (yes vs. no; Yes = moderate, considerable or extreme, No = none or mild); education (trade vs. secondary vs. tertiary); health insurance (uninsured vs. insured); smoking status (current or former smoker vs. never smoked); have close confidante (no vs. yes); mental health history (yes vs. no), number of SCADs (one vs. two or more), time since most recent SCAD (previous year vs. 13–36 months vs. 37–60 months vs. over 5 years ago); attend CR = attend cardiac rehabilitation (no vs. yes). CI = confidence intervals; *p* = significance level. Bold indicates statistically significant results using Benjamini-Hochberg Adjusted p values.

As shown in Table 2, financial strain was a significant independent predictor of higher scores on all domains, while being aged under 50 and having a trade-level education were significant independent predictors of higher scores on all but the *Health system* domain.

#### Lifestyle impacts

The variables retained on statistical grounds for inclusion in the logistic regression to predict lifestyle impacts were age, sex, education, financial strain, living alone, presence of a close confidante, diabetes, obesity, number of SCADs, and time since SCAD. The variables retained on conceptual grounds were mental health history, number of SCADs, time since SCAD, and CR attendance. The variables that did not qualify for inclusion in the multivariate analyses were country of birth, marital status, employment status, recent bereavement, health insurance, smoking status, the remaining CVD risk factors (apart from diabetes and obesity), and each of the SCAD treatments and comorbidities. Results of the multivariable logistic regression are shown in Table 3.

**Table 3:** Predictors of SCAD lifestyle impacts.

	**Stopped working**			**Reduced your work hours**			**Changed jobs**		
	Beta	95% CI	*p*	Beta	95% CI	*p*	Beta	95% CI	*p*
**Age group**									
30–50	0.11	[0.04,0.33]	**< .001**	0.48	[0.23,1.00]	0.051	1.08	[0.48,2.43]	0.844
51–60	0.25	[0.12,0.53]	**< .001**	0.81	[0.44,1.51]	0.506	2.37	[1.20,4.71]	**0.013**
61+	Ref		.	Ref		.	Ref		.
**Sex**									
Male	Ref		.	Ref		.	Ref		.
Female	3.28	[0.34,31.93]	0.307	0.69	[0.22,2.17]	0.526	6.46	[0.78,53.23]	0.083
**Living arrangement**									
Live alone	Ref		.	Ref		.	Ref		.
Live with others	0.88	[0.37,2.07]	0.767	1.15	[0.54,2.43]	0.714	2.18	[0.88,5.40]	0.094
**Financial strain**									
No	Ref		.	Ref		.	Ref		.
Yes	1.65	[0.80,3.39]	0.174	0.83	[0.46,1.49]	0.531	1.56	[0.85,2.87]	0.151
**Education**									
Secondary	1.01	[0.38,2.67]	0.985	0.20	[0.07,0.55]	**0.002**	0.52	[0.22,1.25]	0.145
Trade or TAFE	2.02	[0.94,4.35]	0.072	1.65	[0.89,3.06]	0.109	0.93	[0.47,1.82]	0.834
University	Ref		.	Ref		.	Ref		.
**Have confidante**									
No	1.65	[0.60,4.58]	0.334	1.60	[0.72,3.57]	0.251	2.48	[1.07,5.76]	0.035
Yes	Ref		.	Ref		.	Ref		.
**Mental health history**									
No	Ref		.	Ref		.	Ref		.
Yes	0.70	[0.33,1.51]	0.368	1.07	[0.60,1.90]	0.829	1.29	[0.70,2.38]	0.417
**Attend CR**									
No	Ref		.	Ref		.	Ref		.
Yes	1.31	[0.66,2.61]	0.437	1.04	[0.61,1.76]	0.893	0.73	[0.42,1.27]	0.268
**Diabetes**									
No	Ref		.	Ref		.	Ref		.
Yes	0.54	[0.05,5.43]	0.600	0.67	[0.10,4.45]	0.678	0.94	[0.13,6.64]	0.952
**Obesity**									
No	Ref		.	Ref		.	Ref		.
Yes	1.65	[0.59,4.61]	0.339	1.14	[0.46,2.83]	0.785	0.81	[0.30,2.15]	0.666
**Number of SCAD**									
One	Ref		.	Ref		.	Ref		.
Two or more	3.09	[1.46,6.55]	**0.003**	2.41	[1.28,4.51]	**0.006**	1.23	[0.63,2.40]	0.554
**Time since SCAD**									
In previous year	Ref		.	Ref		.	Ref		.
13–36 months ago	0.92	[0.31,2.74]	0.879	0.97	[0.42,2.22]	0.933	4.06	[1.22,13.44]	0.022
37–60 months ago	0.77	[0.24,2.42]	0.653	0.74	[0.31,1.78]	0.499	4.93	[1.46,16.63]	**0.010**
Over 5 years ago	1.08	[0.37,3.14]	0.881	0.77	[0.34,1.76]	0.542	5.85	[1.79,19.17]	**0.004**
n	292			292			292		
McKelvey and Zavoina R^2^	0.262			0.155			0.211		

	**Sought financial support**			**Ceased doing your favorite sport or exercise**		
	Beta	95% CI	*p*	Beta	95% CI	*p*
**Age group**						
30–50	1.85	[0.46,7.42]	0.382	1.43	[0.72,2.84]	0.313
51–60	2.13	[0.60,7.53]	0.243	0.85	[0.46,1.57]	0.608
61+	Ref		.	Ref		.
**Sex**						
Male	Ref		.	Ref		.
Female	2.14	[0.21,22.10]	0.521	0.49	[0.15,1.64]	0.250
**Living arrangement**						
Live alone	Ref		.	Ref		.
Live with others	1.03	[0.28,3.75]	0.964	2.13	[0.96,4.71]	0.062
**Financial strain**						
No	Ref		.	Ref		.
Yes	5.66	[2.18,14.71]	**< .001**	1.57	[0.89,2.76]	0.121
**Education**						
Secondary	0.64	[0.12,3.41]	0.604	1.10	[0.52,2.34]	0.805
Trade or TAFE	1.40	[0.53,3.70]	0.502	1.83	[0.99,3.40]	0.056
University	Ref		.	Ref		.
**Have confidante**						
No	1.42	[0.43,4.73]	0.565	2.07	[0.93,4.63]	0.076
Yes	Ref		.	Ref		.
**Mental health history**						
No	Ref		.	Ref		.
Yes	0.73	[0.28,1.88]	0.516	0.96	[0.55,1.69]	0.900
**Attend CR**						
No	Ref		.	Ref		.
Yes	3.45	[1.16,10.24]	0.026	1.08	[0.64,1.80]	0.776
**Diabetes**						
No	Ref		.	Ref		.
Yes	3.09	[0.41,23.44]	0.274	1.11	[0.20,6.19]	0.902
**Obesity**						
No	Ref		.	Ref		.
Yes	1.36	[0.35,5.26]	0.653	0.96	[0.39,2.35]	0.921
**Number of SCAD**						
One	Ref		.	Ref		.
Two or more	1.68	[0.62,4.57]	0.313	2.15	[1.16,3.96]	**0.015**
**Time since SCAD**						
In previous year	Ref		.	Ref		.
13–36 months ago	0.43	[0.12,1.53]	0.194	0.91	[0.40,2.11]	0.835
37–60 months ago	0.27	[0.07,1.07]	0.062	0.48	[0.20,1.16]	0.103
Over 5 years ago	0.32	[0.09,1.14]	0.079	0.75	[0.33,1.70]	0.488
n	292			292		
McKelvey and Zavoina R^2^	0.338			0.128		

Note. Full multivariable linear regression models for each rating included: age (under 50 vs. 51–60 vs. 61+), sex (M vs F), living arrangements (alone vs. with others), financial stress (yes vs. no; Yes = moderate, considerable or extreme, No = none or mild); education (trade vs. secondary vs. tertiary); have close confidante (no vs. yes); mental health history (yes vs. no), attend CR = attend cardiac rehabilitation (no vs. yes), have diabetes (yes vs. no), have obesity (yes vs. no), number of SCADs (one vs. two or more), time since most recent SCAD (previous year vs. 13–36 months vs. 37–60 months vs. over 5 years ago); CI = confidence intervals; *p* = significance level. Bold indicates statistically significant results using Benjamini-Hochberg Adjusted p values.

As shown in Table 3, regarding work, the significant independent predictors of: ‘stopping work’ were being aged over 60, and having had more than one SCAD; of ‘reducing work hours’ were having a trade-level or university education, and having more than one SCAD; and of ‘changing jobs’ was being aged 51–60. The significant independent predictor of ‘seeking financial support’ was having financial strain, and of ceasing a favorite sport or exercise was having had more than one SCAD.

### Association between SCAD Impacts and current anxiety and depression

#### Psychosocial impacts

Associations between the Psychosocial Impact domain scores and current anxiety and depression status are shown in Table 4.

**Table 4:** Association between SCAD Psychosocial Impact domains and current anxiety and depression: Differences in mean scores.

SCAD Psychosocial Impact domains ^a^	Overall (*N* = 290) Mean (SD)	Current anxiety status *(N* = 290)				Current depression status (*N* = 287)			
		Anxious *N* = 60 Mean	Not anxious *N* = 230 Mean	*F*	*p*	Depressed *N* = 60 Mean	Not depressed *N* = 227 Mean	*F*	*p*
Emotional impacts	13.38 (6.28)	**17.15**	12.45	29.05	< .001	**17.28**	12.40	31.63	< .001
Concerns about the future	9.36 (4.00)	**12.00**	8.70	36.12	< .001	**11.25**	8.88	17.44	< .001
Difficulties managing SCAD	12.43 (5.17)	**15.67**	11.63	32.05	< .001	**15.58**	11.64	30.47	< .001
Uncertainty and confusion	8.18 (3.60)	**9.83**	7.77	16.44	< .001	**9.73**	7.77	14.72	< .001
Impacts on relationships	6.82 (4.25)	**9.25**	6.22	26.23	< .001	**10.00**	6.03	48.04	< .001
Impacts on self-perception	6.54 (3.12)	**8.65**	5.99	38.95	< .001	**8.62**	6.01	37.49	< .001
Impacts on roles	9.70 (5.75)	**13.20**	8.79	30.87	< .001	**13.67**	8.66	40.44	< .001
Impacts of the health system	5.61 (4.27)	**7.52**	5.11	15.82	< .001	**7.92**	4.98	24.32	< .001

*N* = 290.

^a^ in the six months after the most recent SCAD.

Note. SCAD = spontaneous coronary artery dissection; GAD7 = Generalised Anxiety Disorder 7-item; Anxious = GAD7≥10, Not anxious = GAD7<10. PHQ9 = Patient Health Questionnaire-9-item; Depressed = PHQ9≥10, Not depressed = PHQ9<10. Bold indicates significantly higher scores using Benjamini-Hochberg Adjusted p values.

As shown in Table 4, ratings were highest for the domains of *Emotional Impacts*, *Difficulties Managing SCAD*, and *Impacts on Roles*, and were lowest for the domains of *Impacts on Self-Perception* and *Impacts of the Health System*. Scores on all domains were significantly associated with current anxiety and depression status, with respondents classified as either anxious or depressed scoring significantly higher on all Psychosocial Impact domains than those classified as non-anxious and non-depressed respectively.

#### Lifestyle impacts

Associations between the lifestyle impacts and current anxiety and depression status are shown in Table 5.

**Table 5:** Association between SCAD Lifestyle impacts and current anxiety and depression: Differences in proportions.

SCAD Lifestyle Impacts ^a^	Overall (*N* = 290) n (%)	Current anxiety status *(N* = 290)				Current depression status (*N* = 287)			
		Anxious *N* = 60 n (%)	Not anxious *N* = 230 n (%)	χ2	*p*	Depressed *N* = 60 n (%)	Not depressed *N* = 227 n (%)	χ2	*p*
Stopped work	57 (19.7)	18 (30.0)	39 (17.0)	5.13	.024	15 (25.0)	42 (18.5)	1.26	.262
Reduced work hours	112 (38.6)	27 (45.0)	85 (37.0)	1.30	.254	29 (48.3)	82 (36.1)	2.98	.084
Changed jobs	88 (30.3)	16 (26.7)	72 (31.3)	0.48	.487	21 (35.0)	65 (28.6)	0.92	.338
Sought financial support	28 (9.7)	8 (13.3)	20 (8.7)	1.17	.279	12 (**20.0**)	15 (6.6)	9.99	.002
Ceased doing favorite sport or exercise	130 (44.8)	41 (**68.3**)	89 (38.7)	16.90	< .001	38 (**63.3**)	91 (40.1)	10.36	.001

*N* = 293.

^a^ since most recent SCAD.

Note. SCAD = spontaneous coronary artery dissection; GAD7 = Generalised Anxiety Disorder 7-item; Anxious = GAD7≥10, Not anxious = GAD7<10. PHQ9 = Patient Health Questionnaire-9-item; Depressed = PHQ9≥10, Not depressed = PHQ9<10. Bold indicates significantly higher percentages using Benjamini-Hochberg Adjusted p values.

As shown in Table 5, having ceased doing a favorite sport or exercise was significantly more likely in those who were currently anxious and/or depressed, compared to those who were not. Seeking financial support was significantly more common in those who were currently depressed.

## Discussion

Building on our earlier qualitative study, which identified a broad range of psychosocial impacts of SCAD [18], the present study quantifies these impacts in a sample of around 300 SCAD survivors. Of all the psychosocial impacts assessed, shock about having a heart attack was the most common experience, reported by almost all participants. This is not surprising as SCAD is unexpected and occurs without warning in apparently ‘healthy’ and relatively young individuals with few CVD risk factors who are not seen as typical heart attack candidates [6, 7]. While emotional shock may subside after an initial adjustment period, it can lead to post-traumatic stress and other serious psychological outcomes [14, 29]. Indeed, the traumatic components that can trigger PTSD include the abruptness of the cardiac event, the risk of death, and a sense of loss of control during and after the event [29], all of which are highly relevant for SCAD survivors, as evidenced here.

Worries and concerns about having or indeed triggering another SCAD were the next most common experiences. Given the high recurrence rate [1, 30, 31], fear of re-events is a valid and rational concern for SCAD survivors. Indeed, this fear appears to be more common after SCAD than after other cardiac events: in an earlier study of 194 non-SCAD cardiac patients, in which we used similar data collection procedures, 48% reported fear of having another event [32], lower than the 80% in the present study. As we have noted previously, SCAD survivors need to be given opportunities to express these fears and have them acknowledged and validated [18].

Inextricably linked to fears of triggering SCAD recurrence, uncertainty and confusion about safe and recommended levels of exercise and physical activity were also common. Again the rate was higher than after non-SCAD cardiac events [32]. Previous studies have demonstrated that SCAD survivors commonly receive inadequate or insufficient information regarding safe exercise levels [11, 18, 33, 34], explaining the confusion and uncertainty around this issue. Recent evidence shows that SCAD survivors want tailored physical activity advice to suit individual needs, capabilities and preferences [33]. The need for SCAD-specific CR programs and increased SCAD awareness in general CR programs has also been strongly recommended [16, 18, 33], as has the need for guidelines on resuming physical activity after SCAD [16].

For many participants, concerns about physical activity translated into hypervigilance and avoidance behaviours. Three in four participants reported being overly aware of bodily sensations, and almost one in two had ceased doing a favorite sport or exercise. It is likely that the high level of sadness about having SCAD and feelings of loss of one’s previous lifestyle were, at least in part, a result of these lifestyle restrictions. Hypervigilance to heart-related symptoms and avoidance of triggering activities are common components of both heart-focused anxiety [35, 36] and PTSD [37], and result in reduced exercise capacity and diminished quality of life [35].

Other uncertainties were also common, including uncertainty about life ahead and about managing SCAD. Uncertainty has been a major theme in qualitative studies with SCAD survivors [18, 38]. Indeed, the challenge of navigating uncertainty has been identified as a major determinant of quality-of-life in illness generally [39] and in cardiac illnesses specifically [40, 41]. Uncertainty is heightened in illnesses with unclear management guidelines and progression trajectories [39], hence its particular relevance following SCAD.

Future-related fears were also common. With more than half the participants reporting it, fear of dying appears to be more prevalent after SCAD than after other cardiac events, where it is reported by 33% [32]. With SCAD survivors typically being younger women with family and caregiving responsibilities, the possibility of death may be particularly concerning. Future-related fears in cardiac patients, and the specific fear of death, are associated with medication non-adherence, reduced physical activity and reduced adherence to other cardioprotective behaviours [42], as well as increased likelihood of developing post-event anxiety, depression and PTSD, hence their relevance to cardiac prognosis [42, 43].

A range of other experiences are also notable. Impacts on self-perception including reduced self-confidence and concerns about weight gain were common, as were feelings of loss of control, frustration about fatigue, and concerns about side effects of medications. Over half the participants were also left worrying about the impacts of their SCAD on family members. For almost one in three participants SCAD had impacted on their working life, while one in 10 had sought financial support. These concerns and impacts all contribute to SCAD patients’ sense of loss of autonomy, which involves having reduced control over one’s behaviours and reduced choices in life [44]. Importantly, loss of autonomy reduces effective self-management and compromises emotional wellbeing [44], underscoring the importance of these concerns and losses.

Items on the *Impact of health system* domain were the least commonly endorsed. While over half the participants reported concern that health professionals did not understand SCAD, endorsement of other related items was relatively low. This contrasts somewhat with findings from our qualitative study, which highlighted lack of information provision and support from health professionals as a strong theme [18], and underscores the importance of using mixed research methods involving both qualitative and quantitative approaches. With continuing health professional education, it is possible that SCAD survivors will be better supported in their health-system encounters in the future. Indeed, the 2023 ESC guidelines for the management of acute coronary syndromes highlight considerable progress in the identification of SCAD and increasing consensus regarding its optimal management [5].

The present study also identified the survivor characteristics associated with an increased likelihood of experiencing high impacts after SCAD. Across almost all psychosocial domains, survivors aged under 50 experienced greater psychosocial impact than did their older counterparts, a pattern that is also seen following non-SCAD AMI [45–49]. Those experiencing financial strain were also at increased risk of psychosocial impacts, again mirroring findings seen in studies of non-SCAD AMI patients [32, 45, 46]. A recent study also identified financial strain as a predictor of recurrent cardiac events in women [50], further highlighting its negative prognostic importance. Having a trade-level education was predictive of both psychosocial and work-related impacts, again consistent with non-SCAD cardiac studies [49, 51–55].

Recurrent SCAD appears to increase the risk of some specific impacts. Having more than one SCAD was predictive of increased work impacts, including stopping work and/or reducing work hours, and increased likelihood of ceasing a favorite sport or exercise. Given that engagement in work and maintenance of exercise both contribute to physical and mental health and wellbeing during midlife [56, 57], these additional risks for people who have experienced SCAD recurrence are notable. These findings point to the additional loss of autonomy, control and choice associated with SCAD recurrence, highlighting SCAD recurrence as a particularly stressful experience [44].

The present study has also provided some evidence that experiencing these psychosocial and lifestyle impacts of SCAD increases the risk of later anxiety and depression. Higher scores on each of the eight psychosocial impact domains was associated with increased likelihood of being anxious or depressed at the time of study participation, which varied from less than one to over six years post-SCAD. Having ceased a favorite sport or exercise was similarly associated with later anxiety and depression, while seeking financial support was associated with later depression. While these associations are not necessarily causal, they point to the possibility that early psychosocial impacts of SCAD, and lifestyle restrictions, may lead to anxiety and depression. Post-cardiac event anxiety and depression are associated with reduced quality-of-life and increased risk of recurrent events and premature mortality [58–60], underscoring their prognostic importance.

### Limitations

The present study has some limitations. First, the study sample appears to be relatively well-educated, most likely a result of being drawn from the larger VC Genetics Study sample, therefore it may not be representative of all SCAD survivors. Importantly though, the response rate is very high, decreasing the likelihood of participation bias in this psychosocial sub-study. Indeed, the study sample of approximately 300 is larger than most previous studies in this area. Second, based entirely on self-reporting, there is no objective verification of participants’ current anxiety and depression status, a limitation that is typical of studies of this type. Online self-report might also introduce bias due to non-completion by people with limited vision, dyslexia and other conditions. Third, participants were required to report retrospectively on the psychosocial and lifestyle impacts that had occurred in the six months after their most recent SCAD. For some, this was over 6 years prior to study participation, which may compromise the reliability of responses. It is possible that those with current anxiety or depression may respond more negatively when reflecting on past experiences, thereby artificially increasing associations between mood and SCAD-related experiences. Fourth, we did not collect data on contraceptive use, history of pregnancy, childbirth, pregnancy-related illnesses, or menopause status, therefore we were unable to account for the possible impacts of these potentially confounding factors on psychosocial and lifestyle impacts or anxiety and depression status. Similarly we did not include a measure of job responsibility level, which might have helped to provide a more nuanced understanding of the impacts of SCAD on job changes. Future studies could seek to investigate the impacts of these factors. Finally, we did not include a comparison sample of either non-SCAD AMI patients or age/sex matched healthy individuals, therefore we are unable to fully attribute the experiences reported here as being attributed SCAD exclusively. Future studies could seek to compare the relative rates of specific fears and concerns in SCAD vs. non-SCAD AMI patients, and in age/sex matched non-medical individuals. This would provide a more reliable assessment of the attribution of these concerns to the SCAD event.

### Conclusion and implications

The current study contributes to our understanding of the emotional and lifestyle impacts of SCAD. The findings suggest that SCAD survivors have unmet needs in relation to accessing both informational and emotional support, and point to the need for support strategies in specific areas. First, SCAD survivors may benefit from tailored psychosocial support in the form of evidence-based psychological programs, to assist them in adjusting to and accepting their condition, with particular attention to managing fears and uncertainties regarding SCAD recurrence and the future more generally. Group programs may be particularly beneficial to enhance a sense of camaraderie, safety, empowerment and hope [34, 61]. Support in adjusting to the shock of SCAD may help to ameliorate the risks of PTSD [29]. Second, SCAD survivors need clear and reliable information about how to best self-manage their condition, with a particular emphasis on both generalized guidelines and individually-tailored advice regarding return to physical activity, exercise and sport, and other physically-demanding activities, as previously recommended [16, 18, 33, 34]. Third, survivors may need support to continue working after SCAD if they choose, including flexibility regarding reduced hours and modified tasks. In the face of the unpredictability of SCAD, maintenance of personal autonomy appears to be a particularly important aspect of recovery for SCAD survivors. Targeting of support to those who are younger, with lower education and greater financial stress, and to those who have experienced a second or subsequent SCAD appears to be warranted.

## Supporting information

S1 File (DOCX)
