# Correction: Psychosocial and lifestyle impacts of spontaneous coronary artery dissection: A quantitative study

**DOI:** 10.1371/journal.pone.0304537

**Published:** 2024-05-30

**Authors:** Barbara M. Murphy, Michelle C. Rogerson, Michael R. Le Grande, Stephanie Hesselson, Siiri E. Iismaa, Robert M. Graham, Alun C. Jackson

Fig 1 is incorrect. Domains 1–6 are missing in the published figure. Please see the correct Fig 1 here.

**Fig 1:**
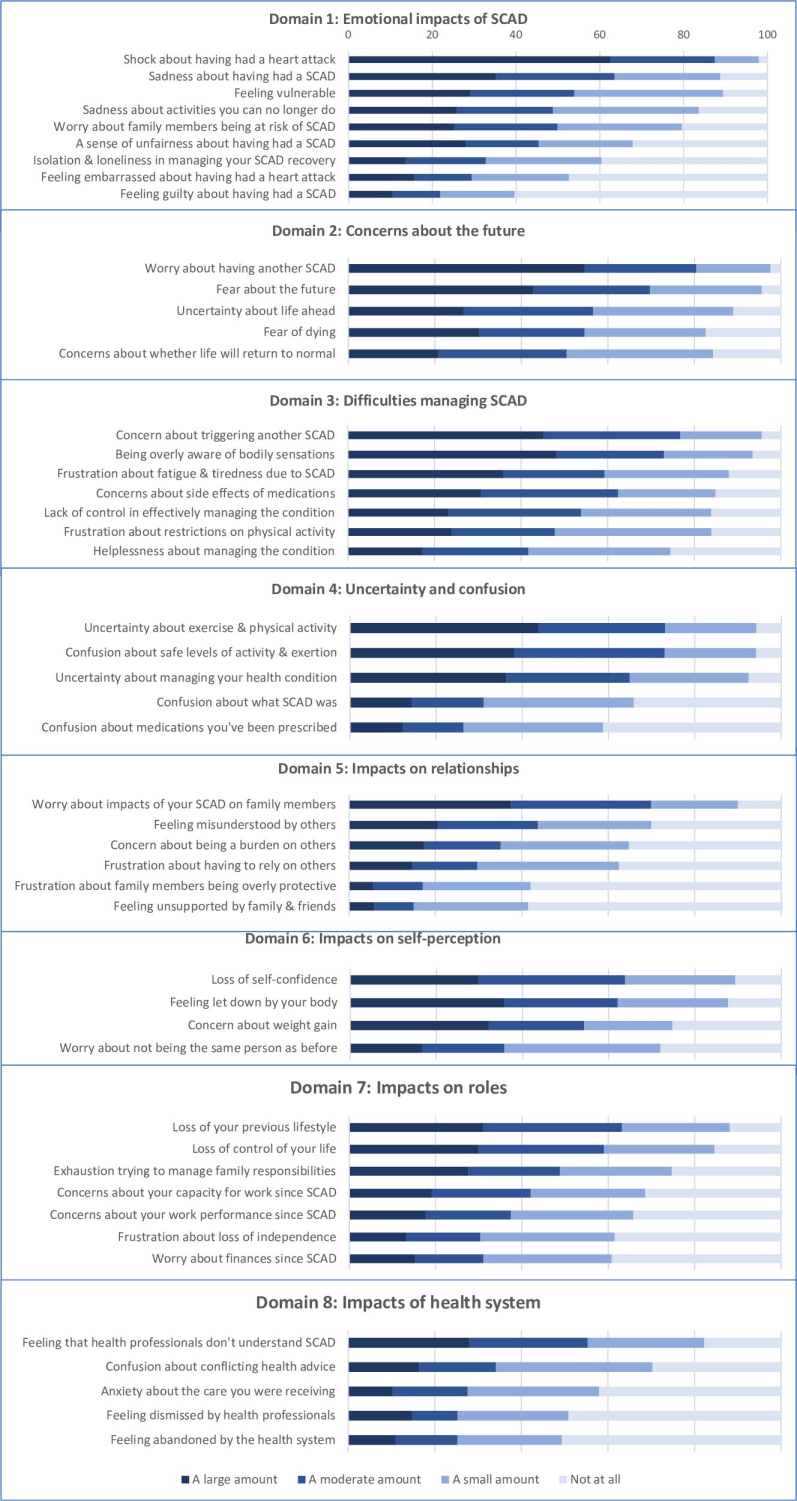
Ratings for all SCAD psychosocial impact items across the eight domains. *N* = 293. Note: SCAD = Spontaneous coronary artery dissection.
