# Supplementary material for: Psychosocial and lifestyle impacts of spontaneous coronary artery dissection: A quantitative study
Source: PLoS One. 2024 Jan 5;19(1):e0296224. doi: 10.1371/journal.pone.0296224 (PMC10769080; doi:10.1371/journal.pone.0296224)
Supplement: S1 File — (DOCX) [file pone.0296224.s001.docx]

**Demographic Questionnaire**

| For each of the following questions, please select or write the answer(s) that best applies to you: | |
| --- | --- |
| **Your age (in years) _________** | |
|  | |
| **Your gender** | |
|  | 1 = Male |
|  | 2 = Female |
|  | 3 = Prefer not to say |
|  | 4 = Prefer to self-describe |
|  |  |
| **Your country of birth** | |
|  | 1 = Australia |
|  | 2 = United Kingdom |
|  | 3 = New Zealand |
|  | 4 = China |
|  | 5 = India |
|  | 6 = Philippines |
|  | 7 = Vietnam |
|  | 8 = Italy |
|  | 9 = South Africa |
|  | 10 = Malaysia |
|  | 11 = Sri Lanka |
|  | 12 = Other ­­­­­­­­­­­­­­­­­­­­­­___________________________ |
|  |  |
| **Are you of Aboriginal or Torres Strait Islander origin?** | |
|  | 0 = No |
|  | 1 = Yes, Aboriginal |
|  | 2 = Yes, Torres Strait Islander |
|  | 3 = Yes, both Aboriginal and Torres Strait Islander |
|  |  |
| **Your current postcode _________** | |
|  |  |
| **Your marital status** | |
|  | 1 = Never married  2 = Widowed  3 = Divorced or separated  4 = Married or living with partner |
|  |  |
| **Your living arrangements** | |
|  | 0 = Live alone  1 = Live with others (with whom?) __________________________________________ |
|  |  |
| **Do you have a close confidante? (someone you can talk to about how you’re feeling)** | |
|  | 0 = No |
|  | 1 = Yes |
|  |  |
| **Do you know other people who have had a SCAD?** | |
|  | 0 = No |
|  | 1 = Yes |
|  |  |
| **Have you lost an important relative or friend over the past year?** | |
|  | 0 = No |
|  | 1 = Yes |
|  |  |
| **Your highest educational level** | |
|  | 1 = Primary |
|  | 2 = Secondary |
|  | 3 = Trade or TAFE qualification |
|  | 4 = University diploma/degree/post-graduate |
|  |  |
| **Your employment status prior to your SCAD** | |
|  | 1 = Employee |
|  | 2 = Self-employed |
|  | 3 = Unemployed |
|  | 4 = Not in the paid workforce (e.g. home duties, voluntary work, retired) |
|  | 5 = Other (specify) _____________________________­­­_________________ |
|  |  |
| **Do you have private health insurance?** | |
|  | 0 = No |
|  | 1 = Yes |
|  |  |
| **What is your current level of financial strain?** | |
|  | 0 = None |
|  | 1 = Slight |
|  | 2 = Moderate |
|  | 3 = Considerable |
|  | 4 = Extreme |
|  | |
| **What is your current smoking status?** | |
|  | 0 = Non-smoker |
|  | 1 = Former smoker (i.e., quit in the past 4 weeks) |
|  | 2 = Current smoker |
|  |  |
| **Have you been told by a doctor or specialist that you had a SCAD?** | |
|  | 0 = No |
|  | 1 = Yes |
|  |  |
| **If yes, was this diagnosis confirmed by a coronary angiogram?** | |
|  | 0 = No |
|  | 1 = Yes |
|  | 2 = I don’t know |
|  |  |
| **How long ago was your most recent SCAD? ________months ________years** | |
|  |  |
| **How many SCADs have you had in total? (drop down menu)** | |
|  | 1 2 3 4 5 or more |
|  | |
| **Are you on medication to manage your SCAD?** | |
|  | 0 = No |
|  | 1 = Yes |
|  |  |
| **To manage your SCAD, have you had any of the following? (select all that apply)** | |
|  | 1 = Stent procedure (percutaneous coronary intervention; PCI) |
|  | 2 = Bypass surgery (coronary artery bypass surgery) |
|  | 3 = Other procedure (specify) _____________________­­­_________________ |
|  |  |
|  | |
| **In the year prior to your SCAD, were you pregnant or did you give birth? (select only females aged less than 50)** | |
|  | 0 = No |
|  | 1 = Yes |
|  |  |
| **Have you ever been diagnosed with any of the following conditions? (select all that apply)** | |
|  | 1 = Fibromuscular Dysplasia (FMD) |
|  | 2 = Connective tissue disorder (e.g., Ehlers-Danlos syndrome, Marfan syndrome) |
|  | 3= Migraine |
|  | 4 = Inflammatory disorder (e.g., lupus, multiple sclerosis, rheumatoid arthritis) |
|  | 5 = High blood pressure |
|  | 6 = Diabetes (Type 1 or 2) |
|  | 7 = Gestational diabetes |
|  | 8 = High cholesterol |
|  | 9 = Obesity |
|  | 10 = Anxiety |
|  | 11 = Depression |
|  | 12 = Post-traumatic stress |
|  | 13 = Stroke or Transient Ischaemic Attack (TIA) |
|  | 14 = Musculoskeletal condition (e.g., arthritis, chronic back pain, osteoporosis) |
|  | 15 = Sleep apnoea |
|  | 16 = Other **___________________________________________________** |
|  |  |
| **Have you been diagnosed with a mental health condition in the past, prior to your SCAD? (select all that apply)** | |
|  | 0 = No |
|  | 1 = Anxiety |
|  | 2 = Depression |
|  | 3 = Other mental health condition (specify) ______________________ |
|  |  |
| **Did you attend cardiac rehabilitation (CR) after your most recent SCAD?** | |
|  | 0 = No |
|  | 1 = Yes |
|  |  |
| **If you did not attend CR, what was your main reason for not attending?** | |
|  | 1 = I chose not to/not relevant to me |
|  | 2 = I was advised not to by my health professional |
|  | 3 = Not available |
|  | 4 = Not referred |
|  | 5 = Other (specify) ______________________ |
|  |  |
| **If you did attend CR, how many sessions did you attend? (drop down menu)** | |
|  | 1 2 3 4 5 6 7 8 9 10 11 12 |
|  |  |
| **Are you part of any of the following? (select all that apply)** | |
|  | 1 = Face-to-face support group |
|  | 2 = Online support group |
|  | 3 = Facebook group |
|  | 4 = Other |
|  | 5 = None of the above |
|  |  |
| **How did you find out about this SCAD survey?** | |
|  | 1 = Victor Chang Cardiac Research Institute (St Vincent’s Hospital Sydney) |
|  | 2 = Australian Centre for Heart Health |
|  | 3 = SCAD alliance |
|  | 4 = Other |
|  |  |

**SCAD Distress Questions**

During the first six months after your most recent SCAD, to what extent did you experience the following:

|  |  | Not at all | A small amount | A moderate amount | A large amount |
| --- | --- | --- | --- | --- | --- |
| 1 | Feeling dismissed by health professionals | 0 | 1 | 2 | 3 |
| 2 | Fear about the future | 0 | 1 | 2 | 3 |
| 3 | Uncertainty about managing your health condition | 0 | 1 | 2 | 3 |
| 4 | Loss of control of your life | 0 | 1 | 2 | 3 |
| 5 | Shock about having had a heart attack | 0 | 1 | 2 | 3 |
| 6 | Loss of self-confidence | 0 | 1 | 2 | 3 |
| 7 | Worry about impacts of your SCAD on family members | 0 | 1 | 2 | 3 |
| 8 | Feeling abandoned by the health system | 0 | 1 | 2 | 3 |
| 9 | Worry about having another SCAD | 0 | 1 | 2 | 3 |
| 10 | Uncertainty about exercise and physical activity | 0 | 1 | 2 | 3 |
| 11 | Loss of your previous lifestyle | 0 | 1 | 2 | 3 |
| 12 | Sadness about having had a SCAD | 0 | 1 | 2 | 3 |
| 13 | Feeling your body has let you down | 0 | 1 | 2 | 3 |
| 14 | Worry about family members being at risk of SCAD | 0 | 1 | 2 | 3 |
| 15 | Confusion about what SCAD was | 0 | 1 | 2 | 3 |
| 16 | Fear of dying | 0 | 1 | 2 | 3 |
| 17 | Confusion about safe levels of activity & exertion | 0 | 1 | 2 | 3 |
| 18 | Lack of control in effectively managing the condition | 0 | 1 | 2 | 3 |
| 19 | Concerns about side effects of medications | 0 | 1 | 2 | 3 |
| 20 | Sadness about activities you can no longer do | 0 | 1 | 2 | 3 |
| 21 | A sense of unfairness about having had a SCAD | 0 | 1 | 2 | 3 |
| 22 | Feeling misunderstood by others | 0 | 1 | 2 | 3 |
| 23 | Feeling unsupported by family and friends | 0 | 1 | 2 | 3 |
| 24 | Confusion about conflicting health advice | 0 | 1 | 2 | 3 |
| 25 | Uncertainty about life ahead | 0 | 1 | 2 | 3 |
| 26 | Concern about triggering another SCAD | 0 | 1 | 2 | 3 |
| 27 | Helplessness about managing your condition | 0 | 1 | 2 | 3 |
| 28 | Frustration about loss of independence | 0 | 1 | 2 | 3 |
| 29 | Feeling guilty about having had a SCAD | 0 | 1 | 2 | 3 |
| 30 | Feeling vulnerable | 0 | 1 | 2 | 3 |
| 31 | Exhaustion trying to manage family responsibilities | 0 | 1 | 2 | 3 |
| 32 | Anxiety about the care you were receiving | 0 | 1 | 2 | 3 |
| 33 | Worry about not being the same person as before | 0 | 1 | 2 | 3 |
| 34 | Frustration about restrictions on physical activity | 0 | 1 | 2 | 3 |
| 35 | Confusion about medications you’ve been prescribed | 0 | 1 | 2 | 3 |
| 36 | Concerns about your capacity for work since having SCAD | 0 | 1 | 2 | 3 |
| 37 | Isolation and loneliness in managing your SCAD recovery | 0 | 1 | 2 | 3 |
| 38 | Frustration about having to rely on other people | 0 | 1 | 2 | 3 |
| 39 | Frustration about family members being overly protective | 0 | 1 | 2 | 3 |
| 40 | Feeling that health professionals don’t understand SCAD | 0 | 1 | 2 | 3 |
| 41 | Concerns about whether life will return to normal | 0 | 1 | 2 | 3 |
| 42 | Frustration about fatigue and tiredness due to SCAD | 0 | 1 | 2 | 3 |
| 43 | Being overly aware of physical sensations in your body | 0 | 1 | 2 | 3 |
| 44 | Concerns about your work performance since having SCAD | 0 | 1 | 2 | 3 |
| 45 | Concern about being a burden on others | 0 | 1 | 2 | 3 |
| 46 | Worry about finances due to your changed circumstances | 0 | 1 | 2 | 3 |
| 47 | Feeling embarrassed about having had a heart attack | 0 | 1 | 2 | 3 |
| 48 | Concern about weight gain | 0 | 1 | 2 | 3 |

Since having your most recent SCAD, have you**:**

|  | Yes | No |
| --- | --- | --- |
| Stopped working | 1 | 0 |
| Reduced your work hours | 1 | 0 |
| Changed jobs | 1 | 0 |
| Sought financial support | 1 | 0 |
| Ceased doing your favourite sport or exercise | 1 | 0 |
| Reconsidered pregnancy or having further children | 1 | 0 |

**What do you believe triggered your SCAD event? (select all that apply):**

Emotional stress in the days leading up to the SCAD Yes/No

Emotional stress in the months leading up to the SCAD Yes/No

Strenuous exercise on the day you developed the SCAD Yes/No

Strenuous exercise in the 7 days prior to developing the SCAD Yes/No

Childbirth/pregnancy in the year prior to developing the SCAD Yes/No

Other (please specify) Yes/No

**Generalised Anxiety Disorder Questionnaire (GAD-7)**

Over the last 2 weeks, how often have you been bothered by any of the following problems? Select the answer that best describes how you have been feeling over the past two weeks.

|  |  | Not at all | Several days | More than half the days | Nearly every day |
| --- | --- | --- | --- | --- | --- |
| Feeling nervous, anxious or on edge? |  | 0 | 1 | 2 | 3 |
| Not being able to stop or control worrying? |  | 0 | 1 | 2 | 3 |
| Worrying too much about different things? |  | 0 | 1 | 2 | 3 |
| Trouble relaxing? |  | 0 | 1 | 2 | 3 |
| Being so restless that it is hard to sit still? |  | 0 | 1 | 2 | 3 |
| Becoming easily annoyed or irritable? |  | 0 | 1 | 2 | 3 |
| Feeling afraid as if something awful might happen? |  | 0 | 1 | 2 | 3 |

**Patient Health Questionnaire (PHQ-9)**

Over the last 2 weeks, how often have you been bothered by any of the following problems? Select the answer that best describes how you have been feeling over the past two weeks.

|  |  | Not at all | Several days | More than half the days | Nearly every day |
| --- | --- | --- | --- | --- | --- |
| Little interest or pleasure in doing things |  | 0 | 1 | 2 | 3 |
| Feeling down, depressed, or hopeless |  | 0 | 1 | 2 | 3 |
| Trouble falling or staying asleep, or sleeping too much |  | 0 | 1 | 2 | 3 |
| Feeling tired or having little energy |  | 0 | 1 | 2 | 3 |
| Poor appetite or overeating |  | 0 | 1 | 2 | 3 |
| Feeling bad about yourself — or that you are a failure or have let yourself or your family down |  | 0 | 1 | 2 | 3 |
| Trouble concentrating on things, such as reading the newspaper or watching television |  | 0 | 1 | 2 | 3 |
| Moving or speaking so slowly that other people could have noticed? Or the opposite — being so fidgety or restless that you have been moving around a lot more than usual |  | 0 | 1 | 2 | 3 |
| Thoughts that you would be better off dead or of hurting yourself in some way |  | 0 | 1 | 2 | 3 |

**Short Form Health Survey -12 (SF-12)**

**Please answer every question by selecting the most appropriate answer.**

**1. In general, would you say your health is:**

_____ Excellent

_____ Very Good

_____ Good

_____ Fair

_____ Poor

**The following two questions are about activities you might do during a typical day.**

**Does your health now limit you in these activities? If so, how much?**

|  | Yes, limited a lot | Yes, limited a little | No, not limited at all |
| --- | --- | --- | --- |
| 2. Moderate activities such as moving a table, pushing a vacuum cleaner, bowling or playing golf | □ | □ | □ |
| 3. Climbing several flights of stairs | □ | □ | □ |

**During the PAST 4 WEEKS have you had any of the following problems with your work or other regular activities as a result of your physical health?**

|  | Yes | No |
| --- | --- | --- |
| 4. Accomplished less than you would like? | □ | □ |
| 5. Were limited in the kind of work or other activities? | □ | □ |

**During the PAST 4 WEEKS, were you limited in the kind of work you do or other regular activities as a result of any emotional problems (such as feeling depressed or anxious)?**

|  | Yes | No |
| --- | --- | --- |
| 6. Accomplished less than you would like? | □ | □ |
| 7. Didn’t do work or other activities as carefully as usual? | □ | □ |

**8. During the PAST 4 WEEKS, how much did pain interfere with your normal work (including both work outside the home and housework)?**

_____ Not At All

_____ A Little Bit

_____ Moderately

_____ Quite A Bit

_____ Extremely

**The next three questions are about how you feel and how things have been DURING THE PAST 4 WEEKS. For each question, please select the one answer that comes closest to the way you have been feeling. How much of the time during the PAST 4 WEEKS –**

|  | All of the time | Most of the time | A good bit of the time | Some of the time | A little of the time | None of the time |
| --- | --- | --- | --- | --- | --- | --- |
| 9. Have you felt calm and peaceful? | □ | □ | □ | □ | □ | □ |
| 10. Did you have a lot of energy? | □ | □ | □ | □ | □ | □ |
| 11. Have you felt downhearted and blue? | □ | □ | □ | □ | □ | □ |

**12. During the PAST 4 WEEKS, how much of the time has your PHYSICAL HEALTH OR EMOTIONAL PROBLEMS interfered with your social activities (like visiting with friends, relatives, etc.)?**

_____ All of the Time

_____ Most of the Time

_____ A Good Bit of the Time

_____ Some of the Time

_____ A Little of the Time

_____ None of the Time

**Cardiac Distress Inventory Short Form (CDI-SF)**

Please indicate whether or not you have experienced each issue during the past four weeks by selecting “Y” for yes or “N” for no. For each item that you have selected “Y”, indicate how much distress this issue has caused you **during the past four weeks** by selecting, on a scale of 0 to 3, where “0” is no distress and “3” is severe distress.

| **Issue** | **Yes** | **No** | **If yes, indicate how much distress this causes for you** | | | |
| --- | --- | --- | --- | --- | --- | --- |
|  |  |  | **No distress at all** | **Slight distress** | **Moderate distress** | **Severe distress** |
| Thinking I will never be the same again | Y | N | 0 | 1 | 2 | 3 |
| Not knowing what the future holds for me | Y | N | 0 | 1 | 2 | 3 |
| Feeling lonely | Y | N | 0 | 1 | 2 | 3 |
| Withdrawing from people | Y | N | 0 | 1 | 2 | 3 |
| Having changes in my usual roles | Y | N | 0 | 1 | 2 | 3 |
| Lacking purpose or meaning in life | Y | N | 0 | 1 | 2 | 3 |
| Being unable to deal with stress | Y | N | 0 | 1 | 2 | 3 |
| Being emotionally exhausted | Y | N | 0 | 1 | 2 | 3 |
| Having difficulty concentrating | Y | N | 0 | 1 | 2 | 3 |
| Being physically restricted | Y | N | 0 | 1 | 2 | 3 |
| Not getting clear directions from my health practitioner on how to manage my heart condition | Y | N | 0 | 1 | 2 | 3 |
| Thinking about dying | Y | N | 0 | 1 | 2 | 3 |

**Fear of Progression Questionnaire short form (FOPQ-SF)**

Below are a list of statements that are related to your illness and possible future concerns. Please select the answer that applies to you.

Some questions may not apply to you. For example, if you are retired, you will not be able to answer the questions about your employment. Please select “never” in these cases.

|  | **Never** | **Seldom** | **Sometimes** | **Often** | **Very often** |
| --- | --- | --- | --- | --- | --- |
| 1. I become anxious if I think my disease may progress | □ | □ | □ | □ | □ |
| 1. I am nervous prior to doctors’ appointments or periodic examinations | □ | □ | □ | □ | □ |
| 1. I am afraid of pain | □ | □ | □ | □ | □ |
| 1. The thought that I might become less productive at my job disturbs me | □ | □ | □ | □ | □ |
| 1. When I am anxious, I have physical symptoms, e.g., rapid heartbeat, stomach- ache, nervousness | □ | □ | □ | □ | □ |
| 1. The possibility of my children contracting my disease disturbs me | □ | □ | □ | □ | □ |
| 1. It disturbs me that I may have to rely on strangers for activities of daily living | □ | □ | □ | □ | □ |
| 1. I am worried that at some point in time, because of my illness I will no longer be able to pursue my hobbies | □ | □ | □ | □ | □ |
| 1. I am afraid of severe medical treatments in the course of my illness | □ | □ | □ | □ | □ |
| 1. I worry that my medications could damage my body | □ | □ | □ | □ | □ |
| 1. I worry about what will become of my family if something should happen to me | □ | □ | □ | □ | □ |
| 1. The thought that I might not be able to work due to my illness disturbs me | □ | □ | □ | □ | □ |

**Mishel’s Uncertainty in Illness Scale (Community version)**

Please read each statement. Then select the answer that most closely measures how you

are feeling TODAY.

Please respond to every statement**.**

|  | Strongly disagree | Disagree | Undecid-ed | Agree | Strongly agree |
| --- | --- | --- | --- | --- | --- |
| I don’t know what is wrong with me | 1 | 2 | 3 | 4 | 5 |
| I have a lot of questions without answers | 1 | 2 | 3 | 4 | 5 |
| I am unsure if my illness is getting better or worse | 1 | 2 | 3 | 4 | 5 |
| It is unclear how bad my symptoms will be | 1 | 2 | 3 | 4 | 5 |
| The explanations they give about my condition seem hazy to me | 1 | 2 | 3 | 4 | 5 |
| The purpose of each treatment is clear to me | 1 | 2 | 3 | 4 | 5 |
| My symptoms continue to change unpredictably | 1 | 2 | 3 | 4 | 5 |
| I understand everything explained to me | 1 | 2 | 3 | 4 | 5 |
| The doctors say things to me that could have many meanings | 1 | 2 | 3 | 4 | 5 |
| My treatment is too complex to figure out | 1 | 2 | 3 | 4 | 5 |
| It is difficult to know if the treatments or medications I am getting are helping | 1 | 2 | 3 | 4 | 5 |
| Because of the unpredictability of my illness, I cannot plan for the future | 1 | 2 | 3 | 4 | 5 |
| The course of my illness keeps changing. I have good and bad days | 1 | 2 | 3 | 4 | 5 |
| I have been given many differing opinions about what is wrong with me | 1 | 2 | 3 | 4 | 5 |
| It is not clear what is going to happen to me | 1 | 2 | 3 | 4 | 5 |
| The results of my tests are inconsistent | 1 | 2 | 3 | 4 | 5 |
| The effectiveness of the treatment is undetermined | 1 | 2 | 3 | 4 | 5 |
| Because of the treatment, what I can do and cannot do keeps changing | 1 | 2 | 3 | 4 | 5 |
| I’m certain they will not find anything else wrong with me | 1 | 2 | 3 | 4 | 5 |
| The treatment I am receiving has a known probability of success | 1 | 2 | 3 | 4 | 5 |
| They have not given me a specific diagnosis | 1 | 2 | 3 | 4 | 5 |
| The seriousness of my illness has been determined | 1 | 2 | 3 | 4 | 5 |
| The doctors and nurses use everyday language so I can understand what they are saying | 1 | 2 | 3 | 4 | 5 |

**UCLA Loneliness Scale - 4-Item Version**

The following statements describe how people sometimes feel. For each statement, please indicate how often you feel the way described. For example: If you were asked the question ‘How often do you feel happy?’, if you never felt happy, you would circle ‘1’, while if you always felt happy, you would circle ‘4’.

|  | Never | Rarely | Sometimes | Always |
| --- | --- | --- | --- | --- |
| 1. How often do you feel that you are “in tune” with the people around you? | 1 | 2 | 3 | 4 |
| 2. How often do you feel that no one really knows you well? | 1 | 2 | 3 | 4 |
| 3. How often do you feel you can find companionship when you want it? | 1 | 2 | 3 | 4 |
| 4. How often do you feel that people are around you but not with you? | 1 | 2 | 3 | 4 |

**ENRICHD Social Support Instrument**

Please read the following questions and select the response that most closely describes your current situation:

|  | None of the time | A little of the time | Some of the time | Most of the time | All of the time |
| --- | --- | --- | --- | --- | --- |
| 1. Is there someone available to whom you can count on to listen to you when you need to talk? | 0 | 1 | 2 | 3 | 4 |
| 2. Is there someone available to you to give you good advice about a problem? | 0 | 1 | 2 | 3 | 4 |
| 3. Is there someone available to you who shows you love and affection? | 0 | 1 | 2 | 3 | 4 |
| 4. Is there someone available to help with daily chores? | 0 | 1 | 2 | 3 | 4 |
| 5. Can you count on anyone to provide you with emotional support (talking over problems or helping you make a difficult decision)? | 0 | 1 | 2 | 3 | 4 |
| 6. Do you have as much contact as you would like with someone you feel close to, someone in whom you can trust and confide in? | 0 | 1 | 2 | 3 | 4 |
| 7. Are you currently married or living with a partner? | YES NO | | | | |

**Illness (SCAD) Identity Questionnaire**

Please indicate how much you agree with each statement, by checking a number from 1 to 5 where 1= strongly disagree and 5=strongly agree.

|  | Strongly disagree | Disagree | Neither | Agree | Strongly agree |
| --- | --- | --- | --- | --- | --- |
| 1. I refuse to see my SCAD as part of myself | 1 | 2 | 3 | 4 | 5 |
| 2. I’d rather not think of my SCAD | 1 | 2 | 3 | 4 | 5 |
| 3. I hate being talked to about my SCAD | 1 | 2 | 3 | 4 | 5 |
| 4. I never talk to others about my SCAD | 1 | 1 | 2 | 3 | 4 |
| 5. I just avoid thinking about my SCAD | 1 | 2 | 3 | 4 | 5 |
| 6. My SCAD simply belongs to me as a person | 1 | 2 | 3 | 4 | 5 |
| 7. My SCAD is part of who I am | 1 | 2 | 3 | 4 | 5 |
| 8. I accept being a person who has had SCAD | 1 | 2 | 3 | 4 | 5 |
| 9. I am able to place my SCAD in my life | 1 | 2 | 3 | 4 | 5 |
| 10. I have learned to accept the limitations imposed by my SCAD | 1 | 2 | 3 | 4 | 5 |
| 11. My SCAD dominates my life | 1 | 2 | 3 | 4 | 5 |
| 12. My SCAD has a strong impact on how I see myself | 1 | 2 | 3 | 4 | 5 |
| 13. I am preoccupied with my SCAD | 1 | 2 | 3 | 4 | 5 |
| 14. My SCAD influences all my thoughts and feelings | 1 | 2 | 3 | 4 | 5 |
| 15. My SCAD completely consumes me | 1 | 2 | 3 | 4 | 5 |
| 16. It seems as if everything I do, is influenced by my SCAD | 1 | 2 | 3 | 4 | 5 |
| 17. My SCAD prevents me from doing what I would really like to do | 1 | 2 | 3 | 4 | 5 |
| 18. My SCAD limits me in many things that are important to me | 1 | 2 | 3 | 4 | 5 |
| 19. Because of my SCAD, I have grown as a person | 1 | 2 | 3 | 4 | 5 |
| 20. Because of my SCAD, I know what I want out of life | 1 | 2 | 3 | 4 | 5 |
| 21. Because of my SCAD, I have become a stronger person | 1 | 2 | 3 | 4 | 5 |
| 22. Because of my SCAD, I realize what is really important in life | 1 | 2 | 3 | 4 | 5 |
| 23. Because of my SCAD, I have learned a lot about myself | 1 | 2 | 3 | 4 | 5 |
| 24. Because of my SCAD, I have learned to work through problems and not just give up | 1 | 2 | 3 | 4 | 5 |
| 25. Because of my SCAD, I have learned to enjoy the moment more | 1 | 2 | 3 | 4 | 5 |

**Patient Activation Scale**

Below are some statements that people sometimes make when they talk about their health. Please indicate how much you agree or disagree with each item.

|  | Strongly disagree | Disagree | Agree | Strongly Agree |
| --- | --- | --- | --- | --- |
| 1. When all is said and done, I am the person who is responsible for managing my SCAD | 1 | 2 | 3 | 4 |
| 2. Taking an active role in my own health care is the most important factor in determining my health and ability to function | 1 | 2 | 3 | 4 |
| 3. I am confident that I can take actions that will help prevent or minimize some symptoms or problems associated with my SCAD | 1 | 2 | 3 | 4 |
| 4. I know what each of my prescribed medications do | 1 | 2 | 3 | 4 |
| 5. I am confident that I can tell when I need to go get medical care and when I can handle my SCAD myself | 1 | 2 | 3 | 4 |
| 6. I am confident I can tell my health care provider concerns I have even when he or she does not ask | 1 | 2 | 3 | 4 |
| 7. I am confident that I can follow through on medical treatments I need to do at home | 1 | 2 | 3 | 4 |
| 8. I understand the nature and causes of my SCAD | 1 | 2 | 3 | 4 |
| 9. I know the different medical treatment options available for my SCAD | 1 | 2 | 3 | 4 |
| 10. I have been able to maintain the lifestyle changes for my health that I have made | 1 | 2 | 3 | 4 |
| 11. I know how to prevent further problems with my SCAD | 1 | 2 | 3 | 4 |
| 12. I am confident I can figure out solutions when new situations or problems arise with my SCAD | 1 | 2 | 3 | 4 |
| 13. I am confident that I can maintain lifestyle changes like diet and exercise even during times of stress | 1 | 2 | 3 | 4 |

**Connor Davidson Resilience Scale**

Please indicate how much you agree with the following statements as they apply to you over the last month. If a particular situation has not occurred recently, answer according to how you think you would have felt.

|  | Not true at all | Rarely true | Some-times true | Often true | True nearly all the time |
| --- | --- | --- | --- | --- | --- |
| 1. I am able to adapt when changes occur | 0 | 1 | 2 | 3 | 4 |
| 2. I can deal with whatever comes my way | 0 | 1 | 2 | 3 | 4 |
| 3. I try to see the humorous side of things when I am faced with problems | 0 | 1 | 2 | 3 | 4 |
| 4. Having to cope with stress can make me stronger | 0 | 1 | 2 | 3 | 4 |
| 5. I tend to bounce back after illness, injury, or other hardships | 0 | 1 | 2 | 3 | 4 |
| 6. I believe I can achieve my goals, even if there are obstacles | 0 | 1 | 2 | 3 | 4 |
| 7. Under pressure, I stay focused and think clearly | 0 | 1 | 2 | 3 | 4 |
| 8. I am not easily discouraged by failure | 0 | 1 | 2 | 3 | 4 |
| 9. I think of myself as a strong person when dealing with life’s challenges and difficulties | 0 | 1 | 2 | 3 | 4 |
| 10. I am able to handle unpleasant or painful feelings like sadness, fear, and anger | 0 | 1 | 2 | 3 | 4 |

**Post-Traumatic Growth Inventory**

Indicate for each of the statements below the degree to which this change occurred in your life as a result of having a SCAD, using the following scale.

0 = I did not experience this change as a result of my SCAD.

1 = I experienced this change to a very small degree as a result of my SCAD.

2 = I experienced this change to a small degree as a result of my SCAD.

3 = I experienced this change to a moderate degree as a result of my SCAD.

4 = I experienced this change to a great degree as a result of my SCAD.

5 = I experienced this change to a very great degree as a result of my SCAD.

|  | No change | Very small degree | Small degree | Mod degree | Great degree | Very great degree |
| --- | --- | --- | --- | --- | --- | --- |
| 1. I changed my priorities about what is important in life. | 0 | 1 | 2 | 3 | 4 | 5 |
| 2. I have a greater appreciation for the value of my own life | 0 | 1 | 2 | 3 | 4 | 5 |
| 3. I have developed new interests | 0 | 1 | 2 | 3 | 4 | 5 |
| 4. I have a greater feeling of self-reliance | 0 | 1 | 2 | 3 | 4 | 5 |
| 5. I have a better understanding of spiritual matters | 0 | 1 | 2 | 3 | 4 | 5 |
| 6. I more clearly see that I can count on people in times of trouble | 0 | 1 | 2 | 3 | 4 | 5 |
| 7. I established a new path for my life | 0 | 1 | 2 | 3 | 4 | 5 |
| 8. I have a greater sense of closeness with others | 0 | 1 | 2 | 3 | 4 | 5 |
| 9. I am more willing to express my emotions | 0 | 1 | 2 | 3 | 4 | 5 |
| 10. I know that I can handle difficulties | 0 | 1 | 2 | 3 | 4 | 5 |
| 11. I can do better things with my life | 0 | 1 | 2 | 3 | 4 | 5 |
| 12. I am better able to accept the way things work out | 0 | 1 | 2 | 3 | 4 | 5 |
| 13. I can better appreciate each day | 0 | 1 | 2 | 3 | 4 | 5 |
| 14. New opportunities are available which wouldn’t have been otherwise | 0 | 1 | 2 | 3 | 4 | 5 |
| 15. I have more compassion for others | 0 | 1 | 2 | 3 | 4 | 5 |
| 16. I put more effort into my relationships | 0 | 1 | 2 | 3 | 4 | 5 |
| 17. I am more likely to try to change things that need changing | 0 | 1 | 2 | 3 | 4 | 5 |
| 18. I have stronger religious faith | 0 | 1 | 2 | 3 | 4 | 5 |
| 19. I discovered that I’m stronger than I thought I was | 0 | 1 | 2 | 3 | 4 | 5 |
| 20. I learned a great deal about how wonderful people are | 0 | 1 | 2 | 3 | 4 | 5 |
| 21. I better accept needing others | 0 | 1 | 2 | 3 | 4 | 5 |

**Thank you very much for completing this questionnaire!**
